# Supplementary figures and images for: Prototype of an organising framework for healthcare decarbonisation research: an exploratory classification study
Source: BMJ Open. 2026 May 7;16(5):e111213. doi: 10.1136/bmjopen-2025-111213 (PMC13157778; doi:10.1136/bmjopen-2025-111213)

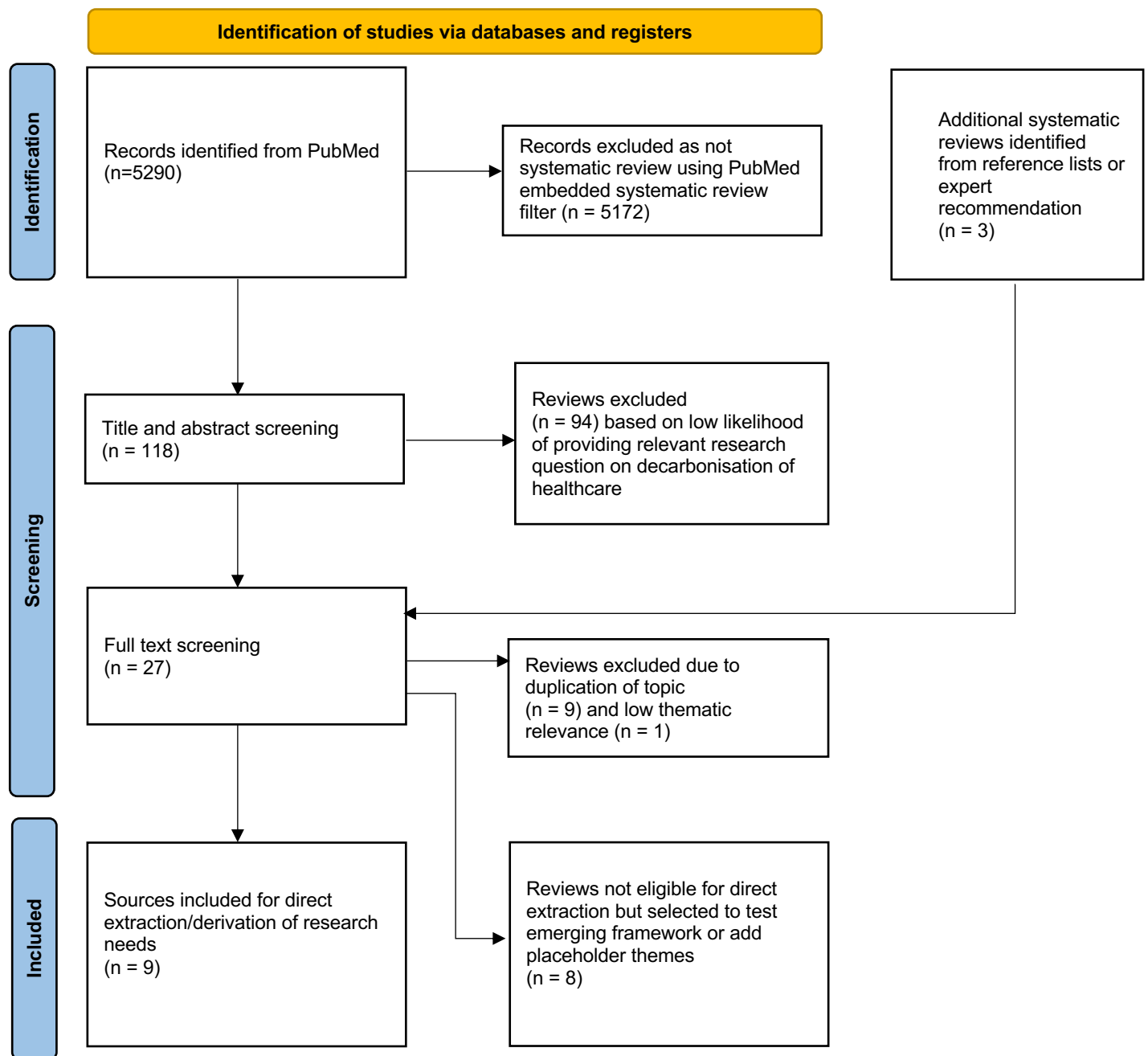

Diagram adapted from Page MJ, et al. BMJ 2021;372:n71. doi: 10.1136/bmj.n71 under license CC BY 4.0.

Supplement: online supplemental figure 1 [file bmjopen-16-5-s001.pdf]
