# Supplementary material for: Prototype of an organising framework for healthcare decarbonisation research: an exploratory classification study
Source: BMJ Open. 2026 May 7;16(5):e111213. doi: 10.1136/bmjopen-2025-111213 (PMC13157778; doi:10.1136/bmjopen-2025-111213)
Supplement: online supplemental file 1 [file bmjopen-16-5-s002.pdf]

Supplement 1: Identification and selection of sources; list of sources used

This supplement first describes the process of identifying and selecting sources (Section A) and then lists the sources used (Section B).

A. IDENTIFICATION AND SELECTION OF SOURCES

Process of narrowing down of preferred sources and forms of research needs
Early discussions within the team revealed a variety of conceptualisations of the guiding concept of 'research needs'*.* Drawing on them and further analytical work by the first author (MP), we generated a working framework of 'forms and sources' of research needs, articulating 12 such forms and sources and over 20 approaches to operationalising the work. We then prioritised six of the forms and sources of research needs and associated approaches to identifying them. After scoping the work, we settled on a process which combined extraction/ derivation of research needs from:

1) **Systematic reviews** concerning health systems decarbonisation retrieved through a pragmatic, highly focused search strategy (see below). These were expected to have identified research gaps through a robust process of locating, mapping, critically evaluating and synthesising already available research.

2) **Key stakeholder sources –** flagship reports or other sources produced by key stakeholders working at the intersection between health and climate change. Such sources were expected to capture 1) knowledge gaps as experienced by a variety of non-academic stakeholders (which may be actual gaps in academic research or indicators of inefficiencies in knowledge dissemination and translation) and 2) knowledge gaps as revealed by the challenges of implementing decarbonisation practices, with the complexity of 'the frontline' and 'the whole' of the health system less likely to be present in academic sources.

2a) Funding calls and recently funded projects were particular subtypes of key stakeholder sources of interest. They were expected to capture a perspective on knowledge gaps that incorporates a cost-benefit analysis. Information on funded projects was also expected to signpost to both research "in the pipeline", which may soon fill in knowledge gaps identified through the other approaches, and highly innovative research which may be making inroads into the "unknown unknowns" of health system decarbonisations.

3) **Pre-existing lists of research needs**, as generated by other initiatives on identifying research/ knowledge gaps and developing research agendas.

Process of identifying sources

Systematic reviews

Systematic reviews concerning health systems decarbonisation were retrieved through a pragmatic, highly focused search strategy in MEDLINE, PubMed interface:

> ("net zero" OR "net 0" OR decarboni* OR sustainab* [ti] OR "climate change" [ti]) AND ("Health Policy"[MAJR] OR "health system" OR healthcare OR "health care")

The goal was to remain at the 'big picture' level (hence the keywords are very broad) and to reduce false positives (hence concepts broader than decarbonisation were only searched for in title and "health policy", aiming to capture research-policy interactions, was only included as a "major heading").

Searches were run in Jun 2024.

At the level of searches, no year, language or country limits were set. To limit to systematic reviews, we applied the embedded PubMed systematic reviews filter. Articles were considered eligible for inclusion if they represented systematic reviews on decarbonisation/ sustainability/ climate change issues at the level of health systems as a whole or at the level of individual components (namely settings, such as hospitals or primary care, or the WHO health system "building blocks", namely leadership and governance; service delivery, health system financing; healthcare workforce; medical products, vaccines and technologies; health

information systems[1]). Articles from healthcare systems of high-income countries were prioritised due to the greater similarity between those systems and the NHS. Screening of articles at title and abstract level was performed by one analyst (MP). Systematic reviews in reference lists or recommended by colleagues were also considered for inclusion.

Supplementary Figure 1 is a PRISMA flow diagram illustrating the identification and screening process used to identify the systematic reviews used as sources.

5,290 publications were retrieved through the search strategy above, of which 118 were systematic reviews. Of these, 24 reviews were selected for further consideration following title and abstract screening. Three further reviews were identified in alternative ways (colleagues' recommendations or reference lists), for an initial shortlist of 27 reviews.

Ultimately, 9 reviews were selected for detailed data extraction (see list of sources below). One review was excluded due to low thematic relevance. Nine reviews were excluded because their topic was already covered by an alternative source. At this exploratory stage, comparing the research recommendations of sources with a significant overlap of coverage was considered low priority. In seven cases (systematic reviews on surgery and operating theatres), the alternative source was the *James Lind Alliance* list [10]. The other two topics for which we had chosen an alternative source were nutrition and digital health.

Eight reviews, from those not included in the direct data extraction were used as background sources to test the emerging organising framework and expand it through placeholder themes. ('Placeholder themes' were themes which were not illustrated by a specific research question, either because there was not enough information to generate a research question from the respective source or because the source was dated; further detail in the manuscript.) Of these, 2 were dated, respectively from 2009 and 2014. Five expanded the organising framework in ways that were fully expected conceptually towards specific medical specialties. One (a rapid review) was accessed after the organising framework had stabilised.

[1] https://extranet.who.int/nhptool/BuildingBlock.aspx

Key stakeholder sources

A list of 30+ key stakeholders was generated and iteratively enriched through suggestions of team members. The key stakeholders were organisations and initiatives which are either:

- firmly positioned at the intersection between healthcare and decarbonisation/ climate change/ environmental sustainability (Type 1 stakeholders), or
- have an influential role in healthcare improvement and can thus be expected to lead and/or enable healthcare decarbonisation (Type 2 stakeholders).

Type 1 stakeholders were primarily UK-based organisations placed in the context of major global actors. Being based in the UK did not, however, seem to be associated with a primary focus on the UK for many of the selected stakeholders, by virtue of climate change being a planetary issue. Type 2 stakeholders were only from the UK.

Box S1a: Key stakeholders, type 1: Organisations with a primary remit at the intersection between healthcare & decarbonisation/ sustainability/ climate change (UK in global context)

UK-centred

Health system structures (for health system structures for Wales and Northern Ireland, see 16 and 17, Box S1b)

1. Greener NHS (NHS England)
2. NHS Sustainability Action (NHS Scotland)

Other country-level structures (not part of the health system governance structure)

3. Green Health Wales
4. Climate Northern Ireland, Health & Wellbeing sector

Non-profit organisations

5. Centre for Sustainable Healthcare
6. UK Health Alliance on Climate Change
7. Sustainable Healthcare Coalition
8. Greener Practice: UK's Primary Care Sustainability Network

Business-oriented networks

9. Climate and Health Coalition (Forum for the Future)

Advocacy and pressure groups

10. Health for Extinction Rebellion (UK)

Global

11. Alliance for Transformative Action on Climate and Health (ATACH)
12. Health Care Without Harm
13. Health and Environment Alliance (HEAL)
14. The Global Climate & Health Alliance

Flagship commissions (UK or global) at the intersection between healthcare & decarbonisation/ sustainability/ climate change

15. Lancet Commission on Sustainable Healthcare

Box S1b: Key stakeholders, type 2: UK organisations driving healthcare quality and improvement (UK only)

Health system structures (for England and Scotland, see 2a)

16. NHS Wales
17. Health and Social Care Northern Ireland

Organisations responsible for setting and/or monitoring compliance with healthcare standards

18. National Institute for Health and Care Excellence (NICE)
19. Care Quality Commission

Networks creating links between the NHS and health research and innovation

20. Health Innovation Network (HIN)
21. National Institute for Health and Care Research Applied Research Collaborations (NIHR ARCs)
22. National Institute for Health and Care Research HealthTech Research Centres (NIHR HRCs)

Think-tanks with a health sector focus

23. The Health Foundation – done, but perhaps leave out for consistency
24. The King's Fund
25. The Nuffield Trust

Communities of practice for healthcare quality and improvement

26. The Academy of Fabulous NHS Stuff

Patient and public involvement organisations

27. Healthwatch
28. The Patients Association
29. NHS PPI groups

Major UK funders with a remit in health

30. National Institute for Health and Care Research (NIHR)
31. The Wellcome Trust
32. UK Research and Innovation (UKRI)
33. Advanced Research + Invention Agency (ARIA)
34. Association of Medical Research Charities (AMRC)
35. NHS Charities Together

Other organisations that have produced research-intense documents at the intersection between healthcare & decarbonisation/ sustainability/ climate change were opportunistically identified.

The preferred type of key stakeholder source was a flagship document (report, paper, strategy, roadmap, etc.) produced or co-produced by a particular organisation, addressing the

decarbonisation of healthcare overall or of the part of the healthcare system that falls within the remit of the organisation (e.g. primary care).

If such a document was not available, any flagship document at the intersection between health/ healthcare & climate change/ the environment was considered. Ideally, the document also dedicated focused attention to the research needed in the domain or had a substantial research underpinning (e.g. was based on an extensive literature review). An exemplar document in terms of attention to research needs (though not a thematic exemplar) was the "Heat Resilience Strategy" of the Physiological Society and Faculty of Public Health [24].

Documents were primarily sought in "Publications" or similar sections of organisational websites. If there was no such section, website pages were reviewed until a document which met the above criteria was found. In some cases, the website copy was used to generate research needs.

Only documents from 2020 onwards were included, to account for the significant changes which healthcare systems have experienced as a result of COVID-19.

Documents or sections within them with a high face validity for relevant contents were read in full. Alternatively (when the table of contents and scanning the whole document did not suggest highly relevant contents, particularly when the document was not solely about healthcare, or to accommodate time constraints towards the end of the project), searches were run for *research*, R&D*, *evaluat*, gap*, knowledge, data, evidence, and method** using the "Find" function of a particular software, typically Adobe.

In the case of funding calls and funded projects – types of key stakeholder sources which differed from the rest in their structure – we reviewed website information on funding calls, lists of funded projects and, at times, funder reports.

Pre-existing standalone lists of research needs

Lists known to the team from before the start of the study were used: of the *James Lind Alliance* on *Greener Operations [10]* and of the *Royal Netherlands Academy of Arts and Sciences (KNAW)* – a *Longlist of knowledge gaps in Planetary Health [11]*. The literature

searching processes for both systematic reviews and key stakeholder documents were expected to capture further standalone lists of research needs, if available.

B. SOURCES USED to generate research questions, by type

Systematic reviews from which research questions were derived (by chronology of data extraction):

1. Keil M, Frehse L, Hagemeister M, et al. Carbon footprint of healthcare systems: a systematic review of evidence and methods. BMJ Open 2024;14:e078464. doi:10.1136/bmjopen-2023-078464
2. Berniak-Woźny J and Rataj M. Towards Green and Sustainable Healthcare: A Literature Review and Research Agenda for Green Leadership in the Healthcare Sector. Int J Environ Res Public Health 2023, 20, 908. https://doi.org/10.3390/ijerph20020908
3. Zurynski Y, Fisher G, Wijekulasuriya S, et al. Bolstering health systems to cope with the impacts of climate change events: A review of the evidence on workforce planning, upskilling, and capacity building. Int J Health Plann Manage 2024;39(3):781-805. doi: 10.1002/hpm.3769.
4. van Schie V. Governance related factors influencing the implementation of sustainability in hospitals: A systematic literature review. Health Policy 2024;146:105115. https://doi.org/10.1016/j.healthpol.2024.105115
5. Pitard M, Rouvière N, Leguelinel-Blache G, Chasseigne V. Contribution of hospital pharmacists to sustainable healthcare: a systematic review. European Journal of Hospital Pharmacy. Online First: 22 May 2024. doi: 10.1136/ejhpharm-2024-004098
6. Pickard Strange M, Booth A, Akiki M, et al. The Role of Virtual Consulting in Developing Environmentally Sustainable Health Care: Systematic Literature Review. J Med Internet Res 2023; 25:e44823. https://www.jmir.org/2023/1/e44823
7. Guillaumie L, Boiral O, Baghdadli A, et al. Integrating sustainable nutrition into health-related institutions: a systematic review of the literature. Can J Public Health 2020;111, 845–861. https://doi.org/10.17269/s41997-020-00394-3
8. Bray L, Meznikova K, Crampton P, Johnson T. Sustainable healthcare education: A systematic review of the evidence and barriers to inclusion. Med Teach 2023;45(2):157-166. doi: 10.1080/0142159X.2022.2110052
9. Braithwaite J, Smith CL, Leask E, et al. Strategies and tactics to reduce the impact of healthcare on climate change: systematic review. BMJ 2024;387:e081284. doi: 10.1136/bmj-2024-081284

Targeted work on identifying knowledge gaps/ research priorities from which research questions were derived:

10. James Lind Alliance. Greener Operations: Sustainable Peri-Operative Practice. Priority Setting Partnership Workshop Outcomes, Jun 2022. Last accessed Feb 2026.
11. Royal Netherlands Academy of Arts and Sciences (KNAW). Longlist of knowledge gaps in Planetary Health. Appendix to the report “Planetary Health. An emerging field to be developed”, 2023. Last accessed Feb 2026.

Key stakeholder reports (12-19) and web sources (20-23) from which research questions were derived:

12. Climate and Health Coalition. Driving Co-benefits for Climate and Health. 2022 Update: How the private sector can accelerate progress. Guidance for businesses, investors and policy makers, Nov 2022. Last accessed Feb 2026.
13. Health Care Without Harm, in collaboration with Arup. Global Road Map for Health Care Decarbonization: A navigational tool for achieving zero emissions with climate resilience and health equity. Health Care Without Harm Climate-Smart Health Care Series. Green Paper Number Two, Apr 2021. Last accessed Feb 2026.
14. NHS England. Delivering a ‘Net Zero’ National Health Service, Jul 2022.
15. NICE (National Institute for Health and Care Excellence). NICE Listens environmental sustainability project recommendations. Appendix to NICE Listens: Public dialogue on environmental sustainability. Final report, Feb 2023. https://www.nice.org.uk/what-nice-does/our-research-work/nice-listens. Last accessed Feb 2026.
16. Scottish Government, NHS Scotland. Climate Emergency & Sustainability Strategy 2022-2026, Aug 2022. Last accessed Feb 2026.
17. The Carbon Trust and GIG Cymru Partneriaeth Cydwasanaethau (NHS Wales Shared Services Partnership). NHS Wales Decarbonisation Strategic Delivery Plan 2021-2030, Mar 2021. Last accessed Feb 2026.
18. UK Health Alliance for Climate Change. End of Year Report 2023, Jan 2024. Last accessed Feb 2026.
19. World Health Organization. Operational framework for building climate resilient and low carbon health systems, Nov 2023. Last accessed Feb 2026.
20. Webpages of the Health for Extinction Rebellion: https://healthforxr.com/in-support-of-health-activism/. Last accessed Feb 2026.
21. Webpages of the Centre for Sustainable Healthcare, case studies: http://networks.sustainablehealthcare.org.uk/CaseStudies. Last accessed Feb 2026.

22. Webpages of NHS England. Greener NHS. System progress, case studies: https://www.england.nhs.uk/greenernhs/whats-already-happening/. Last accessed Feb 2026.
23. Webpages of the Sustainable Healthcare Coalition, on carbon calculators: https://shcoalition.org/care-pathway-carbon-calculator-2/ https://shcoalition.org/coming-soon/. Last accessed Aug 2024 (no longer live Aug 2025).

Other stakeholders' reports from which research questions were generated:

24. The Physiological Society and Faculty of Public Health. Red Alert: Developing a human-centred national Heat Resilience Strategy, Nov 2023. Last accessed Feb 2026.

Funder websites reviewed in detail:

Over 430 funding areas were screened (most on the UKRI website), the majority *not* addressing decarbonisation. Research questions were generated from 2 calls and 2 recently funded projects calls. A number of further calls were used to generate placeholder themes and test the emerging organising framework.

UK Research and Innovation (UKRI)

    Areas of investment and support

    Funding finder

Wellcome

    Climate and Health funding opportunities

    Climate and Health awarded grants

National Institute for Health and Care Research (NIHR)Advanced Research + Invention Agency (ARIA)

Background sources used to generate 'placeholder themes' and test the emerging framework *(organised by reason for exclusion from the pool of sources used to generate research questions):*

Dated but highly relevant systematic reviews:

1. Nichols A, Maynard V, Goodman B, Richardson J. Health, Climate Change and Sustainability: A Systematic Review and Thematic Analysis of the Literature. Environ Health Insights 2009;3:63-88. doi: 10.4137/ehi.s3003

2. McGain F, Naylor C. Environmental sustainability in hospitals - a systematic review and research agenda. J Health Serv Res Policy 2014;19(4):245-52. doi: 10.1177/1355819614534836

Medical specialty-specific systematic reviews:

3. Martin N, Sheppard M, Gorasia G, et al. Awareness and barriers to sustainability in dentistry: A scoping review. Journal of Dentistry 2021;112:103735.
4. Martin N, Sheppard M, Gorasia G, et al. Drivers, opportunities and best practice for sustainability in dentistry: A scoping review. Journal of Dentistry 2021;112:103737.
5. Anudjo MN, Vitale C, Elshami W, et al. Considerations for environmental sustainability in clinical radiology and radiotherapy practice: a systematic literature review and recommendations for a greener practice. Radiography 2023; 29(6):1077-92.
6. Cohen ES, Kouwenberg LH, Moody KS, et al. Environmental sustainability in obstetrics and gynaecology: a systematic review. BJOG: An International Journal of Obstetrics & Gynaecology 2024;131(5):555-67.
7. Spinos D, Doshi J, Garas G. Delivering a net zero National Health Service: where does otorhinolaryngology–head and neck surgery stand? The Journal of Laryngology & Otology 2024;138(4):373-80.

Key stakeholder publications which did not yield research questions:

8. Aumônier S and Collins M for the Sustainable Healthcare Coalition. Healthcare, Circular Economy Principles and Sustainable Wellbeing. Undated, upload Feb 2020. Last accessed Feb 2026.
9. BMJ and UK Health Alliance on Climate Change. Net Zero Clinical Care 2023: Key Summaries Report, Jan 2024. Last accessed Aug 2025.
10. Care Quality Commission. "Environmental sustainability – sustainable development" element of the Single assessment framework of the CQC. Last accessed Feb 2026.
11. McGeoch L, Hardie T, Coxon C and Cameron G for the Health Foundation. Net zero care: what will it take? Sep 2023. Last accessed Feb 2026.
12. Smith R, Stancliffe, R, Clark W, et al. for the UK Health Alliance on Climate Change, Centre for Sustainable Healthcare, Healthcare Without Harm Europe, Sustainable Healthcare Coalition and Health Declares Climate & Ecological Emergency. Six steps to promote recovery of the health and social care system from the covid-19 pandemic. The BMJ Opinion, 24 Sep 2020.

13. UK Health Alliance on Climate Change. Our manifesto for the UK General Election: Five priorities to sustain our health, health and care services, and environment, 2024. Last accessed Feb 2026.
14. WHO. WHO guidance for climate-resilient and environmentally sustainable health care facilities, Oct 2020. Last accessed Feb 2026.

Key stakeholder websites which did not yield relevant documents, but elements of whose contents were used to generate placeholder themes:

15. Greener Practice: The UK's primary care sustainability network [website]. Last accessed Feb 2026.
16. Applied Research Collaborations (ARCs). Webpages listed under: https://www.nihr.ac.uk/about-us/what-we-do/infrastructure/applied-research-collaborations. Last accessed Feb 2026.
17. HealthTech Research Centres (HRCs). Webpages listed under: https://www.nihr.ac.uk/about-us/what-we-do/infrastructure/healthtech-research-centres. Last accessed Feb 2026.
18. Health Innovation Network [website]. Last accessed Feb 2026.

Sources of stakeholders which were excluded from the list of "key stakeholders" once its inclusion criteria were finalised:

19. Sawyer, M. SEE Sustainability. Climate emergency declaration: A guide for primary care, 2021. Last accessed Feb 2026.
20. SEE Sustainability. General practice non-clinical carbon calculator. Last accessed Feb 2026.

Internal University of Cambridge sources (study was co-funded by the University of Cambridge):

21. *University of Cambridge.* Cambridge Zero's Climate Change Research Map. Excel spreadsheet, 2024.

Source reviewed after organising framework had stabilised:

22. Bragge P, Delafosse V, Ngo C, et al. A Rapid Review of Sustainable Healthcare Interventions. The Monash Sustainable Development Institute. Evidence Review Service, Jun 2024. Last accessed Feb 2026.
