# Supplementary material for: Prototype of an organising framework for healthcare decarbonisation research: an exploratory classification study
Source: BMJ Open. 2026 May 7;16(5):e111213. doi: 10.1136/bmjopen-2025-111213 (PMC13157778; doi:10.1136/bmjopen-2025-111213)
Supplement: online supplemental file 2 [file bmjopen-16-5-s003.pdf]

Supplement 2: Content types and transformations

This supplement describes the process of generating research questions from the sources listed in Supplement 1. Research questions could rarely be directly extracted. Instead, they were derived through an interpretative process. Table 2a lists the types of contents used in this process. Table 2b described the types of transformations to which the source content was subjected.

Table S2a: Types of content from which questions could be generated

TYPES OF CONTENTS IN SOURCE DOCUMENTS	DESCRIPTION
**Contents found across source types (systematic reviews and key stakeholder sources)**	
**Research gaps, needs or recommendations**	Statements which explicitly identify a research gap or need or make an explicit recommendation for a type of research to be conducted or an area of research to be prioritised.<br><br>In some cases, the sources we reviewed (systematic reviews or key stakeholder documents) pointed to a research gap or recommendation as expressed in one of the references they themselves had consulted. When there was a clear singular attribution to an external source, we reference both the source in our sample and the respective original source without, however, having accessed the latter.
**Research limitations**	In a systematic review, contents of this type are typically:<br>• statements from the section describing the limitations of the research, whether of the systematic review itself or of the source studies;<br>• statements about risk of bias assessments.<br><br>In policy documents, contents of this type are typically statements that describe limitations of national data.
**Research availability**	Statements that certain topics have been researched to a limited degree unaccompanied by an explicit research recommendation. For instance, a systematic review may state that only two studies address a particular issue yet not suggest that further research on that issue is needed.

TYPES OF CONTENTS IN SOURCE DOCUMENTS	DESCRIPTION
	On the one hand, the research recommendation may be considered implied, at least in a broader list of research recommendations than the one included in the review. On the other hand, an argument can be made that even if the authors identified a research gap, they made a judgement not to prioritise it and that this judgement would apply even if they could broaden their list of research recommendations.
**Contents found in key stakeholder sources only**	
**Research-policy-healthcare-frontline collaboration**	Statements which refer to an interaction between researchers/ research institutions, policy makers and practitioners, with the interaction either 'ongoing' (currently underway) or 'intended' (envisaged as future work).
**Recommendation for evidence-based guidance development**	Statements concerning the development of evidence-based guidance in healthcare. These have been individuated as a separate category as guidance development tends to be underpinned by an infrastructure of close links between policy, practice and academic research.
**"Language acts" with a non-trivial new knowledge component**	Statements in key stakeholder documents which function as verbal actions (such as commitments or a requirements) supposed to be followed by practical actions and which involve research, data, evaluation, or other form of new knowledge. The new knowledge is non-trivial and needs to be generated or shared by specialists (researchers, analysts, evaluators, domain experts), whether in thematic or methodological areas.<br><br>Examples of such language acts are directives, commitments, intentions, or acknowledgements of need.
**Intention to fund or commission research – problem statement**	In the case of funding organisation sources, used for statements which articulate:<br>• a problem or research area which is targeted by a current funding initiative/ call for proposals. The descriptions can be quite broad but also quite specific and technical;<br>• research question(s) or research area, as per the funding proposal of the researcher or team which received the funding. Such work may be in the future or ongoing.

**TYPES OF CONTENTS IN SOURCE DOCUMENTS**	**DESCRIPTION**
	In the case of other key stakeholder documents, used for statements expressing an intention or recommendation to commission a study, with no specific detail of a funding call.

Table S2b: Types of transformations of source contents (used when research questions could not be directly extracted)

TYPE OF TRANSFORMATION	DESCRIPTION
**Decarbonisation focus attributed**	The research need was formulated in terms of healthcare 'decarbonisation' unlike the source statement which could be framed in terms of climate change, sustainability (environmental or broader) or 'being green' in the context of healthcare and/or in interaction with health outcomes.<br><br>Overall, the assumption is that the decarbonisation aspect is entailed in all other concepts, since they are broader than the concept of decarbonisation. It is nonetheless logically possible that while there is a research need at the higher level (e.g. climate change and health), it has been addressed at the lower level (decarbonisation).
**Boundaries of setting (re) specified**	Used when the research need was formulated about the health system (the focus of the current study) or a 'healthcare context' (underspecified), while the setting referred to in the original study was different. It could be broader than that of the health system, e.g. the health sector, or narrower, e.g. hospitals.<br><br>This transposition is largely logically justified when the boundary is narrowed down (from health sector to healthcare system) and logically problematic when the boundary is expanded (from, for instance, hospitals to health systems). In the latter case, we deemed that a question was still worth formulating, with further checks needed if the research need applies to the health system level.<br><br>Priority was given to the research topics as opposed to the settings of the studies which first proposed them. It became clear in the process of the study that most questions can be meaningfully asked for all the levels of the healthcare system.
**Elements individuated or re-combined**	Used when a recommendation for research includes too many elements or where related elements appear in different research recommendations. 'Elements' refers primarily to topics, but research recommendations can also specify study settings, participant types, methods, conceptual 'lenses', etc. The aim has been to keep research questions focused on one main topic, unless a judgement was made that several issues in combination make a more natural study.
**Claims synthesised/ level of abstraction increased**	Used when a research need has been identified in more than one source or in more than one statement within the same source. Then the phrasing and concepts from the different sources needed to be aligned and synthesised. As concepts across statements and sources are often at a different level of abstraction, such synthesis tends to mean that the resulting research need is formulated in more abstract terms than the formulation in at least one of the sources.

	The more abstract concept can be from one of the statements or external to any of them (proposed by the analyst).
**Concept or contents articulated further**	Used when the source statement was clarified or articulated further by adding brief new contents. The goal has been to make the research question more precise, usually in cases when the source statement was not sufficiently clear. As this increases the distance from the source data, the additions are indicated in square brackets [].
**Research perspective added**	Used when stakeholder sources list or showcase multiple exemplars or case studies which, however, remain at the level of the particular. A research theme or question is then added proposing systematic analysis and synthesis of the case studies.
**Meta-science perspective added**	Used when a source statement from a key stakeholder document leads to a research question that probes something about the data collection or analysis methods referred to in the statement. (Meta-science is the practice of science using its methods upon itself, for instance to inspect its methods and improve them or to address research challenges.)<br><br>Note that this transformation is applied if the analyst made a judgement that there is a need for benchmarking against best methodological practices in academic research. If the need for scientific self-reflection is explicit in the source statement, then no such reformulation is needed. In such cases, the question is added under the *Scientific measurement and theory* category of the framework.
**Editing for standalone clarity**	The aim has been to formulate research questions and themes that make sense if written on a card to be discussed in stakeholder workshops or priority setting exercises. This meant that, on occasions, we needed to include contents from the broader text so that referring back to it is not needed if the questions are read without access to the sources.<br><br>Reformulating for standalone format requires primarily to:<br>• Rephrase concepts and shortcuts of expression which are only clear contextually in the source document. In some cases, this is a matter of filling back some missing words and is largely uncontroversial. In other cases, there is a need for interpretation in statement which the analyst perceives as unclear.<br><br>• Ensure (relative) consistency of format. Currently, the approach has been to use an explanatory phrase (representing a broad theme) for the higher- and middle-level gaps and a question format at the most specific level. In some cases, a ‘theme’ format was used at the most specific level too, as the research gap was under-specified in the source.
