# Supplementary material for: Prototype of an organising framework for healthcare decarbonisation research: an exploratory classification study
Source: BMJ Open. 2026 May 7;16(5):e111213. doi: 10.1136/bmjopen-2025-111213 (PMC13157778; doi:10.1136/bmjopen-2025-111213)
Supplement: online supplemental file 3 [file bmjopen-16-5-s004.pdf]

Supplement 3: Principles underpinning the development of the organising framework

This supplement describes the 10 main principles applied in developing and refining the organising framework. These principles entail certain value judgements which need a broader debate. The content of the principles also needs further articulation.

1. Variety of perspectives

The framework recognises that there are different perspectives to the complex field it represents. It seeks to lift them into high-level categories as opposed to committing to a dominant perspective, which, in the context of decarbonisation, is typically that of sources of carbon and other greenhouse gases.

The key advantage of a multi-perspectival approach is that it enables a broad range of users to identify the perspective that best suits their organisational remit, levers of influence, priorities and mental models. Its key disadvantage is that it leads to duplication and redundancy.

2. Some priority of the mental models of policy and decision makers

We have given some priority to the mental models and needs of policy and decision makers, partly resulting from the orientation of the study, partly in recognition of the significant power of such stakeholders to drive change at the level of national healthcare systems.

This priority can be seen in three main choices. First, we have individuated categories for "big picture, cross-cutting issues" even if, logically, the higher-level category already captures them. For instance, there is a category of *A5.1. Transport, travel and mobility for healthcare purposes and in healthcare contexts (big picture, cross-cutting issues)* under the higher-level category of *A5. Transport, travel and mobility for healthcare purposes and in healthcare contexts.* Policy and decision makers are often interested in overviews of research, while some databases of research literature have a default setting of "automatic explosion" (automatically retrieving research on specific topics under the general topic), meaning that overview work is not easy to find if searches are run on the broad theme. Second, we created a high-level category of *Organisational levers for change*, many of which are policy-level

structures and mechanisms. Third, we raised into separate categories issues of trade-offs and value conflicts (see principle 7 for detail).

3. Dynamic reformulations and re-organisation balanced against a need for standardisation and stability

The framework was developed inductively, expanding and refining it as new research questions emerged from the source documents. We reconceptualised categories at the middle level of generality multiple times to accommodate new questions and new perspectives. We settled for the version we are presenting after we reached "light saturation" of themes: new questions continued to emerge from source documents, but they could be accommodated by existing categories. We hypothesise that the saturation we reached was sample-dependent and that broadening the inclusion criteria for document types will lead to multiple new themes, at least at the middle level of generality.

Since the field of healthcare decarbonisation is exceptionally dynamic, we argue that the framework also needs to remain dynamic and to be regularly updated to reflect new developments. However, it can serve its envisaged functions of coordinating research and action and enabling the sharing of knowledge only if stabilised in certain respects and for certain periods of time.

4. Variety of levels of commitment to decarbonisation

Choices about the phrasing of categories reflect an intention to remain open to different levels of endorsement of the agenda for healthcare decarbonisation, including opposition to it. On some occasions, we are explicit about the polarities of a continuum, e.g. in the phrasing of *E1.6. Legal and regulatory enablers and barriers to the decarbonisation of healthcare*. In other cases, we keep the relationship between concepts vague. For instance, the label of *D3.3. Pharmacists and the decarbonisation of healthcare* can accommodate both the action and inaction of pharmacists and various types of relationships between the two concepts and the realities behind them.

We also chose to tone down the use of strongly pro-decarbonisation language, for instance by replacing an earlier phrasing around the "*imperative* to decarbonise healthcare". Strong endorsement of the goal of decarbonisation will, undoubtedly, be dominant at the level of specific sources tagged by concepts from the framework. However, we aimed to generate a

framework which, at the formal level, is hospitable to all perspectives. Denial of climate change and, respectively, of the need for healthcare decarbonisation is a fundamental part of the debate which should be addressed through evidence, not conceptual barriers.

5. User-friendliness, plain language and relative context-independence in formulations within the constraints imposed by the technical language of research

The intention has been to avoid, as much as possible, technical language in formulating categories. For instance, we preferred a phrasing of "Healthcare decarbonisation in the social conversation" as opposed to "Healthcare decarbonisation in the social discourse".

At this stage, we have also chosen to add detail and illustrative examples in the very label of a category in order to clarify its scope as opposed to choosing briefer labels and adding scope notes. The goal has been to make a category sufficiently clear by reading its name only. For example, we have categories such as *B1. Decarbonisation of healthcare spaces (buildings, facilities, estates, sites, etc.)* and *F2.1. "Healthcare decarbonisation" – conceptualisations, boundaries with related concepts, and operationalisations* as opposed to, for instance, *Decarbonisation of healthcare spaces* and *Healthcare decarbonisation, conceptual*.

Whether the primary users of the framework would prefer detail or parsimony requires empirical testing.

Another feature of the prioritisation of user-friendliness concerned the resolution of tensions between hierarchical level in logical terms and hierarchical level in terms of visibility. When a topic received significant attention in the sources but was at a lower (less visible) hierarchical level logically, we raised it in the hierarchy to increase its visibility. This made for a worse classification system from a scientific perspective but for a more user-friendly one.

We also aimed to account for users for whom English is a second language by choosing simpler vocabulary and grammatical structures.

6. Intention to align categories with relevant conceptual models

So far, the organising framework has been developed inductively. Background knowledge of popular conceptual frameworks in healthcare (e.g. the 4 Ss of Space, Stuff, Staff and Systems) or the WHO building blocks of health systems has been used to inform some labels,

but to a limited degree. We see value in incorporating, in future stages of the work, the intellectual effort accumulated in pre-existing frameworks or conceptual/ theoretical analysis.

Such frameworks may already be well standardised and embedded in classification systems used globally, e.g. the MeSH tree for types of healthcare personnel. They may be healthcare system-specific, e.g. national conceptualisations of careers in the health service, such as NHS careers. They may be associated with legal requirements, such as applying the waste hierarchy in England and Wales (DEFRA, Waste Management Plan for England, Aug 2020; p. 10). Conceptual systematic reviews, such as the Pyone et al., 2017 review of 16 frameworks of health system governance, can also enable conceptual refinements of the framework.

7. Attention to trade-offs and value conflicts

Complex interactions and trade-offs have been lifted to higher levels. This reflects a hypothesis that barriers to action on decarbonisation are less a matter of disagreeing about its importance than of complex, at times tragic, dilemmas of using healthcare resources and managing short-term demand vs. long-term needs.

8. Attempt at balancing the formal, transferable and the thematic, domain-specific

An attempt has been made to abstract formal features of approaches as opposed to prioritise their domain-belonging, even though domain-centred categories are also systematically represented. For instance, a research question around using traditional medicines as a guide in drug discovery led to the category of *Combining innovation with a 'back to basics' approach*, as opposed to only generating a drug discovery-related category.

9. Use of the formal to pave the way to synthesis as opposed to reinforcing silos

An attempt has been made to enable a comparative perspective and/or synthesis where contrasts and conflict seem to dominate. The example above of *Combining innovation with a 'back to basics' approach* is relevant here too, or the bringing together of *Top-down and/or bottom-up approaches to driving healthcare decarbonisation.*

10. Recognition of grey zones between healthcare decarbonisation and broader climate and health issues

Case-by-case decisions were made about remaining in decarbonisation boundaries or expanding towards climate change or sustainability. The scope is broadened when a

decarbonisation focus was perceived as artificial relative to the (likely) nature of the practical work and dominant debate. For instance, some of the subthemes under *A3. Waste, reuse, recycling and circularity in healthcare* concern primarily pollution as opposed to decarbonisation. Such boundary or even out-of-scope topics can offer important opportunities to bridge debates which are distinct but also closely inter-related.
