# Supplementary material for: Prototype of an organising framework for healthcare decarbonisation research: an exploratory classification study
Source: BMJ Open. 2026 May 7;16(5):e111213. doi: 10.1136/bmjopen-2025-111213 (PMC13157778; doi:10.1136/bmjopen-2025-111213)
Supplement: online supplemental file 4 [file bmjopen-16-5-s005.pdf]

Supplement 4: Full NHS-SOS framework

A. NATURAL RESOURCE USE AND SOURCES OF CARBON
**A1. Energy sources and water use in healthcare**
A1.1. Energy sources and water use in healthcare (big picture, cross-cutting issues)
A1.2. Non-renewable energy sources [PLACEHOLDER]
A1.3. Renewable energy sources
A1.4. Water use
A1.5. Heating and cooling
A1.6. Lighting
A1.7. Energy use for new digital demands
**A2. Medicines, equipment and consumables as decarbonisation targets; associated production, procurement and supply chains**
A2.1. Medicines, equipment and consumables as decarbonisation targets; associated production, procurement and supply chains (big picture, cross-cutting issues)
A2.2. Medicines as decarbonisation targets
A2.3. Disinfecting and protective consumables and equipment as decarbonisation targets
A2.4. Medical gases as decarbonisation targets
**A3. Waste, reuse, recycling and circularity in healthcare**
A3.1. Management and reduction of healthcare waste (big picture, cross-cutting issues)
A3.2. Innovations in waste management
A3.3. Reducing waste
A3.4. Reuse
A3.5. Recycling
A3.6. Waste from healthcare – by type
A3.6.1. Medical and hazardous healthcare waste
A3.6.2. Wastewater from healthcare facilities
A3.6.3. Plastic waste, clinical [PLACEHOLDER]
**A4. Food and nutrition in healthcare contexts and for health-related outcomes**
A4.1. Stakeholders' knowledge, perceptions and attitudes to sustainable nutrition
A4.2. Polices, guidelines, strategies and plans on sustainable nutrition
A4.3. Networks and communities of practice for sustainable nutrition
A4.4. Complex interventions on sustainable nutrition [PLACEHOLDER]
A4.5. Low-carbon nutrition in interaction with other principles of sustainable nutrition (e.g. health benefits, equality, affordability) [PLACEHOLDER]
A4.6. Shaping sustainable eating behaviours of patients and staff
**A5. Transport, travel and mobility for healthcare purposes and in healthcare contexts**

A5.1. Transport, travel and mobility for healthcare purposes and in healthcare contexts (big picture, cross-cutting issues)
A5.2. The healthcare fleet
A5.3. Staff travel, transport and mobility
A5.4. Patient travel, transport and mobility for the purpose of accessing healthcare [PLACEHOLDER]
B. HEALTHCARE SETTINGS AND WORKFLOWS
**B1. Decarbonisation of healthcare spaces (buildings, facilities, estates, sites, etc.)**
B1.1. Decarbonisation of healthcare spaces (big picture, cross-cutting issues)
B1.2. Decarbonisation of hospitals
B1.3. Decarbonisation of operating theatres
B1.4. Decarbonisation of sterilisation units
B1.5. Decarbonisation of chemotherapy units
**B2. Decarbonisation of healthcare services (primary care, secondary care, community care, etc.) [PLACEHOLDER]**
**B3. Decarbonisation of clinical specialities and/or health conditions**
B3.1. Surgery as a decarbonisation target
B3.2. Asthma care as a decarbonisation target [PLACEHOLDER]
B3.3. Radiology and radiotherapy as decarbonisaton targets
B3.4. Obstetrics and gynaecology as decarbonisation targets
B3.5. Otorhinolaryngology as a decarbonisation target
B3.6. Dentistry as a decarbonisation target
**B4. Decarbonisation of healthcare supply chains**
**B5. Sustainable reorganisation of healthcare systems in context of emergencies, disasters and other stresses and shocks**
C. SOLUTIONS
**C1. Solutions for decarbonising healthcare (big picture, cross-cutting issues)**
**C2. Digital solutions**
**C3. Innovative materials**
**C4. Innovative designs**
**C5. Capture and removal of greenhouse gases [PLACEHOLDER]**
**C6. New models of care and decarbonisation**
**C7. Combining innovation and ‘back to basics’ approaches**
**C8. Improved healthcare efficiency as a route to decarbonisation**
C8.1. Improved prevention
C8.2. Improved diagnosis

C8.3. Minimising the provision of care of limited or no benefit
C8.4. Reducing overprescribing and overuse of medicines
C8.5. Improved chronic disease management
C8.6. Improved healthcare efficiency – by clinical specialty or health condition
**C9. Education, training and information provision**
**C10. Behaviour change-focused solutions for decarbonising healthcare**
**C11. Complex interventions for decarbonising healthcare**
**C12. The carbon footprint of adaptations to climate change and decarbonisation solutions themselves**
C12.1. The carbon footprint of digital health
C12.2. The carbon footprint of increasing the climate resilience of healthcare
C12.3. The carbon footpring of cooling technologies in healthcare
D. STAKEHOLDERS
**D1. Stakeholders in healthcare and/or decarbonisation (big picture, cross-cutting issues)**
**D2. The healthcare workforce and the decarbonisation of healthcare**
D2.1. Healthcare workforce planning and the decarbonisation of healthcare
D2.2. Training, development and capacity building for the decarbonisation of healthcare
D2.3. Healthcare workers' behaviour change for the decarbonisation of healthcare
D2.4. Formal responsibilities and accountability of healthcare workers concerning the decarbonisation of healthcare
D2.5. Healthcare workers' activism on climate change
D2.6. Healthcare workers' needs for psychological support related to climate change
D2.7. Impact on healthcare workers of work environments responding (or not) to climate change
**D3. Types of healthcare workers and the decarbonisation of healthcare**
D3.1. Doctors and the decarbonisation of healthcare [PLACEHOLDER]
D3.2. Nurses and the decarbonisation of healthcare [PLACEHOLDER]
D3.3. Pharmacists and the decarbonisation of healthcare<br>D3.4. Allied health profesionals and the decarbonisation of health care [PLACEHOLDER]
**D4. Patient populations and groups and the decarbonisation of healthcare [PLACEHOLDER]**
E. ORGANISATIONAL LEVERS FOR CHANGE
**E1. Policy and governance for the decarbonisation of healthcare**
E1.1. Policy and governance (big picture, cross-cutting issues)
E1.2. Leadership
E1.3. Stakeholders [See Section D]

E1.4. Embedding principles of healthcare decarbonisation in organisational structures and processes
E1.5. Top-down and/or bottom-up approaches to driving the decarbonisation of healthcare
E1.6. Legal and regulatory enablers and barriers to the decarbonisation of healthcare
E1.7. Targets, pledges and commitments to enable the decarbonisation of healthcare
**E2. Funding and financial mechanisms for the decarbonisation of healthcare**
**E3. Practical guidance for the decarbonisation of healthcare**
F3.1. Case studies of decarbonisation initiatives
F3.2. Toolkits and other ‘how to’ guides for designing and/or implementing decarbonisation initiatives
**E4. Certification and accreditation schemes for healthcare decarbonisation [PLACEHOLDER]**
**E5. Visions, models and frameworks of healthcare provision which incorporate decarbonisation principles**
E5.1. “Realistic medicine” (Scotland)
**E6. Networks, communities of practice and platforms for collaborative work on healthcare decarbonisation**
E6.1. Co-production and co-implementation of decarbonisation research by research, healthcare, business and/or policy actors [PLACEHOLDER]
E6.2. Networks within healthcare working towards decarbonisation
E6.3. Intersectoral collaborations for decarbonisation, with participation from the health sector [PLACEHOLDER]
**E7. Managing (perceived) trade-offs and conflicts of values in decarbonising healthcare**
E7.1. Balancing clinical effectiveness, patient preferences, financial and environmental costs
E7.2. Addressing the challenge of upfront investment now to achieve decarbonisation benefits later [PLACEHOLDER]
E7.3. Values, ethics and human rights in healthcare decarbonisation
E7.4. Decision making and risk management frameworks
F. SCIENTFIC MEASUREMENT AND THEORY
**F1. Measuring the carbon footprint and the decarbonisation of healthcare**
F1.1. Comprehensive methods and tools for calculating carbon footprint
F1.2. Source-of-carbon-specific methods and tools for calculating carbon footprint
F1.3. Methods and tools for calculating carbon footprint as per their overall methodological-theoretical orientation
F1.4. Sensors and sensing systems for monitoring greenhouse gas emissions in healthcare contexts [PLACEHOLDER]
F1.5. Impact measures in healthcare decarbonisation

F1.6. Comparative measures in healthcare decarbonisation
F1.7. Quality standards for data, evidence and metrics on healthcare decarbonisation
F1.8. Integrated monitoring, reporting and evaluation
F1.9. Decarbonisation targets [PLACEHOLDER]
F1.10. Domain-specific issues in decarbonisation measurement
**F2. Concepts, frameworks and theories concerning the decarbonisation of healthcare**
F2.1. “Healthcare decarbonisation” – conceptualisations, boundaries with related concepts, and operationalisations
F2.2. Applied interdisciplinary fields of inquiry with applications in healthcare decarbonisation
F2.2.1. Decision science for healthcare decarbonisation
F2.2.2. Stakeholder theory for healthcare decarbonisation
F2.2.3. Quality improvement frameworks for healthcare decarbonisation
**F3. Healthcare decarbonisation in the social conversation**
**F4. Needs for quality improvement in research on healthcare decarbonisation**
**F5. Systematic approaches to identifying research and gaps in research on healthcare decarbonisation**
**F6. Turning knowledge into action on healthcare decarbonisation**
