# Supplementary material for: Prototype of an organising framework for healthcare decarbonisation research: an exploratory classification study
Source: BMJ Open. 2026 May 7;16(5):e111213. doi: 10.1136/bmjopen-2025-111213 (PMC13157778; doi:10.1136/bmjopen-2025-111213)
Supplement: Supplementary file 6 [file bmjopen-2025-111213.reviewer_comments.pdf]

PEER REVIEW HISTORY

BMJ Open publishes all reviews undertaken for accepted manuscripts. Reviewers are asked to complete a checklist review form and are provided with free text boxes to elaborate on their assessment. These free text comments are reproduced below.

ARTICLE DETAILS

Title (Provisional)

A prototype of an organising framework for healthcare decarbonisation research: an exploratory classification study

Authors

Petrova, Mila; Burrows, Fanny; van der Scheer, Jan; Kipouros, Timoleon; Smith, James

---

VERSION 1 - REVIEW

**Reviewer**	**1**
**Name**	**Spencer, Rachel**
**Affiliation**	**University of Warwick, Warwick Medical School**
**Date**	**07-Jan-2026**
**COI**	**I am funded by the NIHR 153231 to research greener general practice but I think this enhances my ability to review this paper rather than presenting a CoI.**

This work is very timely. I can see the immediate applications of the proposed framework to the NIHR and other funders of climate research to identify priority areas. I can see it being used in the write up of NIHR 153231 which I am part of.

Abstract

Generally reads clearly. In “design” perhaps state more clearly what the additional 21 sources are. In “results” the presentation of grouping is perhaps unnecessary and can be discussed in the main text. The acronym is convenient and hopefully memorable.

If you are able suggest add “planetary health” to your key terms for the paper.

Introduction

Initially slightly alarmed to see only 14 references, but then found the other sources are referenced elsewhere (supp 1). Was this done to avoid any stipulation by the journal? Suggest you push back and ask for them to be included in main ref list.

Published only very recently (perhaps why) but there are none from my colleague Raquel Nunes who has led a number of review publications on greener general practice. E.g. e091404.full.pdf The field is changing fast – consider a rpt search to flesh out your intro/discussion with the latest references.

Methods

We have also found trouble with MeSH terms relating to decarbonisation, really appreciate the discussion of this in the paper.

I read supplement 1 with interest – would have appreciated greater clarity on exclusion criteria that led you from 118 to just 10 literature reviews. PRISMA diagram may help??

Much of the methods complexity is in supplement 2 (I wonder if this is to avoid word count in the main paper). I am happy with what is in supp 2 but perhaps describe in main text just how exhaustive a process you followed (maybe say how many pages supp 2 runs to?).

The readership may struggle to understand: "Philosophically, promiscuous and pluralistic realism acknowledge that while an objective reality exists…" I know I did.

The lack of PPIE is a shame (especially given the implications for research and practice section where you cite using it for co-production)

Results

I think you might generate a very different focus if you designed the framework with patients from the ground up and you might get some push-back when you show them the current version – particularly in A4! In table 1 "D4 – placeholder" is an issue for me – its obviously vital and yet your prior definition of placeholder indicates it might not be.

Discussion

I expected to see more reference to other taxonomies (not just in climate) and their adoption into research/practice.

On reflection on reading the whole paper I think you should adapt the title to include the word "prototype".

**Reviewer**	**2**
**Name**	**Gray, William**
**Affiliation**	**NHS Improvement, Getting It Right First Time**
**Date**	**12-Jan-2026**
**COI**	**None**

This is a well written and useful piece of work that helps to clarify some of the thinking around environmentally sustainable healthcare prioritisation. The resulting framework draws together key themes that can inform future research on decarbonisation in healthcare.

Although the emerging themes are broadly what one might expect, it is useful to have them pulled together in a single organising framework that can be built on in future. Such a framework is needed within this emerging, and currently disorganised, field of research. The paper is very well written and very clear. I only have minor comments.

• It would be good to have more detail in the main document taken from Supplement 2 on the seven types of content identified and the ten principles. Reading the main document, these seven source contents and ten principles come rather out of the blue and without context in the main manuscript. Perhaps add a sentence or two to each to flesh out a little the reasoning behind inclusion. The supplement can still be used to provide further context.

• I am not sure that the two parts of supplement 2 sit well together as they cover different aspects of the methods. Could they be separated into distinct supplements.

• I accept that the authors wished to create a memorable acronym, and this is of value. Although most of the level 1 themes seem well thought through, I struggled with Theme B as 'Healthcare Contexts', which maybe doesn't really convey what the level 2 themes represent. Could the authors justify this a bit more. I feel that the level 2 themes seem to capture 'healthcare decarbonisation settings' or 'carbon producing healthcare activities'.

• Reference 4. Should be Net Zero, not Net 0. The reference is also very similar to reference 7. Would a single reference suffice?

VERSION 1 - AUTHOR RESPONSE

Reviewer: 1<br>*Dr. Rachel Spencer, University of Warwick*	
This work is very timely. I can see the immediate applications of the proposed framework to the NIHR and other funders of climate research to identify priority areas. I can see it being used in the write up of NIHR 153231 which I am part of.	Thank you for the positive words and for already envisaging uses of the paper! We do hope it contributes to debates on funding priorities.
Abstract	
Generally reads clearly. In "design" perhaps state more clearly what the additional 21 sources are.	Thank you for this suggestion. We have clarified as follows:<br><br>*These and another 22 sources that did not meet fully the inclusion criteria (e.g. dated but highly relevant systematic reviews) or met the inclusion criteria but did not yield research questions were used to specify further areas of thematic relevance and test the emerging framework.*<br><br>* The change in number is due to transferring a rapid review to the category of background sources, as, by the time we accessed it, the organising framework had already stabilised. We

	thus used it to test the framework, but not to formulate new research questions.
In "results" the presentation of grouping is perhaps unnecessary and can be discussed in the main text. The acronym is convenient and hopefully memorable.	Thank you for the suggestion – we considered it but decided to leave the figures for the second and third level themes as they signal the granularity of the work at the level of the abstract. We moved this detail, however, after spelling out the acronym. We are glad you thought the acronym worked.
If you are able suggest add "planetary health" to your key terms for the paper.	Thank you for the suggestion, added.
Introduction<br>Initially slightly alarmed to see only 14 references, but then found the other sources are referenced elsewhere (supp 1). Was this done to avoid any stipulation by the journal? Suggest you push back and ask for them to be included in main ref list.	Thank you for the suggestion. We have now integrated the references for all sources used to generate research questions in the main reference list, only leaving the background sources solely in the Supplement.
Published only very recently (perhaps why) but there are none from my colleague Raquel Nunes who has led a number of review publications on greener general practice. E.g. e091404.full.pdf<br>The field is changing fast – consider a rpt search to flesh out your intro/discussion with the latest references.	Thank you for suggesting this highly relevant new reference.<br><br>We have acknowledged this point in the text and as suggested added multiple references, including two by Nunes, to the discussion. Added to end of strength and limitations section:<br><br>*"It is recognised that this field is rapidly evolving, supported by increasing research funding, and relevant research has been published since the work to develop this initial framework was undertaken (e.g. 40-45)."*<br><br>Also added these words to implication to research section so this aligns with preceding text better:<br>"*emerging research"*
Methods	
We have also found trouble with MeSH terms relating to decarbonisation, really appreciate the discussion of this in the paper.	We also appreciate the acknowledgement. The limited number of MeSH terms is additionally concerning since they are a resource of the US National Library of Medicine.
I read supplement 1 with interest – would have appreciated greater clarity on exclusion criteria that led you from 118 to just 10 literature reviews. PRISMA diagram may help??	Thank you for this query about important methodological detail. We have now added to Supplement 1 the full list of health system building blocks, as per the WHO model, which we considered relevant (namely leadership and governance; service delivery, health system

	financing; healthcare workforce; medical products, vaccines and technologies; health information systems[1]). We prioritised reviews concerning the healthcare system level, the level of the building blocks, and setting types (e.g. community or hospital). As this was a scoping-like review of a pragmatic nature, we have not recorded the specific figures under each exclusion category when moving from the list of 118 to 24. Specific exclusion figures are listed (in the Supplement again) for the reduction of the list from 27 systematic reviews (24 + 3 recommendations) to 9 (as noted above, we have now transferred a rapid review to the list of background sources to better reflect its use, changing the figure from 10 to 9). By memory, the greatest number of exclusions was because of health system type as per income band of countries (low, low middle and middle income).<br><br>Because of the limitations of the underpinning small scoping-like review in terms of recording exclusions, we do not believe that a PRISMA diagram will be sufficiently informative to include one.<br><br>We have adjusted the language in the abstract and methods of main paper to 'pragmatic scoping-like review' to clarify that the process of gathering initial sources was not a full scoping review.
Much of the methods complexity is in supplement 2 (I wonder if this is to avoid word count in the main paper). I am happy with what is in supp 2 but perhaps describe in main text just how exhaustive a process you followed (maybe say how many pages supp 2 runs to?).	Thank you for this comment. We considered its spirit and integrated it with Comment 18 of the second reviewer, suggesting we include detail about the seven types of source contents and the 10 principles from Supplement 2 into the main text. Hopefully the revision strikes a better balance of representing relevant methodological detail and not detracting too much from the focus of the paper, which is the organising framework.
The readership may struggle to understand: "Philosophically, promiscuous and pluralistic realism acknowledge that while an objective reality exists…" I know I did.	Thank you for highlighting. We have now edited as follows. We hope it is an improvement, even if this is never going to be a natural sentence for the primary audience we are writing for:<br><br>*The philosophical frameworks of promiscuous and pluralistic realism (which are highly influential in the philosophy of biology but remain largely*

[1] https://extranet.who.int/nhptool/BuildingBlock.aspx

	unfamiliar in the philosophy of evidence-based medicine) emphasise, amongst other issues, that there are multiple valid ways to classify species and objects, as opposed to a single best and/or most objective way.
The lack of PPIE is a shame (especially given the implications for research and practice section where you cite using it for co-production)	We fully agree on the importance of patient and public engagement. We did this work so as to ground meaningful engagement of stakeholders, including patients and the public. We have added the following sentence to the Limitations sections – second sentence here, the first (in grey) left to provide context:<br><br>*These principles and their combination do, however, entail value judgements which merit further debate and articulation in the process of future co-production work on the organising framework. Considering them from a patient and public perspective is particularly important. None of the documents included in the data extraction represented this perspective in sufficient detail, even though patient and public involvement organisations' websites were included in the search for sources.*
Results<br>I think you might generate a very different focus if you designed the framework with patients from the ground up and you might get some push-back when you show them the current version – particularly in A4! In table 1 "D4 – placeholder" is an issue for me – its obviously vital and yet your prior definition of placeholder indicates it might not be.	Thank you for this comment. We partly addressed it in the way outlined above, partly by revising some of the representation of "placeholders". As it stood, there was a strong implication that they may not reflect a robust research need due to dated information. However, placeholders were also added when the underpinning contents was not enough to formulate a specific enough research question. We have now revised the sentence in question to:<br><br>*Placeholder themes are clearly labelled as such to indicate that a theme needs further evidence and debate to articulate current or emerging research questions and determine its relative priority.*
Discussion<br>I expected to see more reference to other taxonomies (not just in climate) and their adoption into research/practice.	Thank you for this suggestion. We did consider it seriously, as literature on taxonomies, ontologies and classification systems is very relevant to this study. We ultimately decided against adding more on this issue as we thought

	a substantial paragraph would be needed to do the subject justice and this would break the current flow of the Discussion, add length to an already long paper, and add a technical complexity which readers might find challenging (similar to comment 12 above).<br><br>There is already a brief indication that further research should also consider "developments in classification theory and domain ontologies, and user experience research on how people interact with classification or organising systems".
On reflection on reading the whole paper I think you should adapt the title to include the word "prototype".	Thank you for this suggestion. We have now revised the title to: ***A prototype of an organising framework for healthcare decarbonisation research: an exploratory classification study***<br><br>Thank you once again for the encouragement and helpful comments. We hope we have addressed them adequately.
Reviewer: 2<br>*Dr. William Gray, NHS Improvement*	
This is a well written and useful piece of work that helps to clarify some of the thinking around environmentally sustainable healthcare prioritisation. The resulting framework draws together key themes that can inform future research on decarbonisation in healthcare. Although the emerging themes are broadly what one might expect, it is useful to have them pulled together in a single organising framework that can be built on in future. Such a framework is needed within this emerging, and currently disorganised, field of research. The paper is very well written and very clear. I only have minor comments.	Thank you for the positive feedback! We hope that, indeed, the framework will be built upon and support greater coherence of healthcare decarbonisation research and its more effective translation into practical applications.
It would be good to have more detail in the main document taken from Supplement 2 on the seven types of content identified and the ten principles. Reading the main document, these seven source contents and ten principles come rather out of the blue and without context in the main manuscript. Perhaps add a sentence or two to each to flesh out a little the reasoning behind inclusion. The supplement can still be used to provide further context.	Thank you for this suggestion. We have now added 1-2 sentences for each of the seven types of contents and ten principles.
I am not sure that the two parts of supplement 2 sit well together as they cover different aspects of the methods. Could they be separated into distinct supplements.	Thank you for the suggestion. We have now split supplement 2 into two supplements; keeping together the types of sources and types of transformations in supplement 2, and moving the 10 principles used to develop the organising

	framework into a new supplement 3. It certainly makes for more logical documents.
I accept that the authors wished to create a memorable acronym, and this is of value. Although most of the level 1 themes seem well thought through, I struggled with Theme B as ‘Healthcare Contexts’, which maybe doesn’t really convey what the level 2 themes represent. Could the authors justify this a bit more. I feel that the level 2 themes seem to capture ‘healthcare decarbonisation settings’ or ‘carbon producing healthcare activities’.	Thank you for querying, as we had hesitations of whether to go for simplicity of the heading or better correspondence with lower levels. We have changed the heading of this theme to Healthcare Settings and Workflows. The missing ‘workflow’ element explains why some of the sub-themes sound like ‘activities’ (though the idea is that these activities are generally integrated into longer and relatively stable sequences). We wanted to include both spatial and temporal contexts in one category, as they often overlap – e.g. certain spatial contexts reflect temporal sequences, temporal sequences in providing healthcare have spatial implications, etc.<br><br>Because we are now using the word ‘settings’ for the top level theme, we have changed the name of subtheme B2 to ‘decarbonisation of healthcare services’ rather than ‘…settings’.
• Reference 4. Should be Net Zero, not Net 0. The reference is also very similar to reference 7. Would a single reference suffice?	Thank you for noticing, changed from 0 to Zero. We have removed reference 7.<br><br>Thank you once again for the positive feedback and helpful suggestions, particularly in terms of finding a better balance between providing relevant methodological detail and not overwhelming readers with it.

VERSION 2 - REVIEW

**Reviewer**	**1**
**Name**	**Spencer, Rachel**
**Affiliation**	**University of Warwick, Warwick Medical School**
**Date**	**09-Mar-2026**
**COI**	

check the grammar in the "design" part of the abstract

I don't think describing the work as pragmatic gets round the concerns I had about methods

https://www.prisma-statement.org/scoping there is specific guidance on how to add PRSIMA diagrams to scoping reviews - my strong recommendation that you follow this.

PPIE comments were not really addressed

Discussion comments were not really addressed

Reviewer **2**

Name **Gray, William**

Affiliation **NHS Improvement, Getting It Right First Time**

Date **17-Mar-2026**

COI

Thank you for providing this revised manuscript. All my queries have been addressed. The Healthcare Settings and Workflows theme works well.

VERSION 2 - AUTHOR RESPONSE

No	Editor or Reviewer Comment	Authors' responses
	Reviewer: 1<br>*Dr. Rachel Spencer, University of Warwick*	
4b	Check the grammar in the "design" part of the abstract	We have rewritten the design part of the abstract to simplify it and include a verb in the first sentence.<br><br>Previous version: **"Design:** An exploratory manual (non-machine-led) classification study of over 160 research questions derived from a pragmatic scoping-like review of 9 systematic reviews (118 screened), 13 stakeholder documents (35 key stakeholder websites searched), two research priority exercises, and four research funder sources (over 430 funding areas screened). These and another 22 sources that did not meet fully the inclusion criteria (e.g. dated but highly relevant systematic reviews) or met the inclusion criteria but did not yield research questions were used to specify further areas of thematic relevance and test the emerging framework."<br><br>New version: "**Design:** An exploratory classification study of over 160 research questions derived from a review and data extraction of 9 systematic reviews, 13 stakeholder documents, 2 research

No	Editor or Reviewer Comment	Authors' responses
		priority exercises, and 4 research funder sources. A further 8 systematic reviews and 14 stakeholder documents, which were not used for direct data extraction, were used to test the emerging framework and specify thematic gaps."
10b	I don't think describing the work as pragmatic gets round the concerns I had about methods https://www.prisma-statement.org/scoping there is specific guidance on how to add PRSIMA diagrams to scoping reviews - my strong recommendation that you follow this.	The majority of this has been addressed in supplement 1 where as recommended, we have added a PRISMA diagram as Supplementary Figure 1.<br><br>We have also rearranged and slightly amended the section of systematic review identification in supplement 1 to clarify the numbers involved and process used.<br><br>One systematic review had erroneously been included in the previous count of systematic reviews used to test the framework, although not included in the list in Supplement 1 so this was removed from the count and added to the count of those excluded at full text review. Where needed the main text, as well as the supplement, has been amended to reflect this.<br><br>In the main paper we have:<br>- simplified the description of the review undertaken in the main paper from 'pragmatic scoping-like review' to simply 'review'. We felt this was appropriate given that this is not the main focus of the paper, which is a classification study, and as the methods are fully described in Supplement 1.<br>- added a few words in the section on source documents to explain why exclusions were made and to explain that 35 stakeholder websites were used to identify 13 stakeholder reports and web sources.<br>- the sources used to test the emerging framework and placeholders themes were further described as '8 systematic reviews and 14 stakeholder documents which were not used for direct data

No	Editor or Reviewer Comment	Authors' responses
		extraction' rather than just '22 sources'. This aligns the language in this section with the changes to the abstract described above.
13b	PPIE comments were not really addressed.<br><br>Original comment: The lack of PPIE is a shame (especially given the implications for research and practice section where you cite using it for co-production)	We have amended and rearranged the previously suggested text to strengthen the acknowledgement on the importance of PPI and expand on how it could be included at the next stage of development as follows.<br><br>In the PPIE section:<br><br>Previous version:<br>"There was no direct patient and public involvement in this study. It is recognised that patients are key stakeholders in the healthcare system and as such their input will be sought in future developments of the framework."<br><br>Updated text:<br>"There was no direct patient and public involvement in this study. Patient and public involvement organisations' websites were examined but did not generate sources of research questions. Furthermore, patient perspectives were sought within the sources reviewed but none of those included in the data extraction represented this perspective in detail. It is recognised that patients are key stakeholders in the healthcare system and as such their input will be sought in future developments of the framework, building on the prototype generated through this study."<br><br>And in the Discussion:<br>Previous version: "Considering them* from a patient and public perspective is particularly important. None of the documents included in the data extraction represented this perspective in sufficient detail, even though patient and public involvement organisations'

No	Editor or Reviewer Comment	Authors' responses
		websites were included in the search for sources."<br>* refers to previous sentence about principles used to developing themes.<br><br>Updated text: "A limitation of the initial development of this prototype classification framework was that as a desk-based exercise this process did not include patient and public participation. It is planned that patients will be involved in the next stage of development including reviewing the prototype, critiquing the emerging structure and themes, advising on usefulness and potential uses, and informing its future development."
15b	Discussion comments were not really addressed<br><br>Original comment on discussion: I expected to see more reference to other taxonomies (not just in climate) and their adoption into research/practice.	We have now added to and amended the discussion section to include the following text with reference to other taxonomies and their adoption into research/practice:<br><br>Previous version:<br>"Growing and refining the organising framework will also benefit from engaging with research and expertise concerning its formal structure – such as developments in classification theory and domain ontologies, and user experience research on how people interact with classification or organising systems."<br><br>Updated text:<br>"The development of the NHS-SOS framework as a prototype of a classification system of research questions contributes to the field of health- and research-related classification, taxonomy and ontology development. Biomedical ontologies are numerous, with 1,275 included in BioPortal, the most comprehensive repository of biomedical ontologies, as of March 2026.(45) In the field of research classifications, the Health Research Classification System, developed by the UK Clinical Research

No	Editor or Reviewer Comment	Authors' responses
		Collaboration on the basis of the WHO International Classification of Diseases and the Common Scientific Outline, has been used both by UK and international health research funders to classify and strategically assess award portfolios.(46) The rise of information technology and AI requires increasingly complex classification systems and allows for non-manual methods of generating these. Bibliometric analysis of technology in sustainable healthcare is an example of the use of non-manual methods to describe the rapidly evolving field of technology in healthcare.(47) Growing and refining the NHS-SOS organising framework requires its mapping against and partial or full integration with relevant domain ontologies. The work would also benefit from engagement with research and expertise concerning user interactions with classification systems and new developments in classification theory."<br><br>This resulted in the addition of three references related to this field (references 45-47).
